# Effects of TiO_2_ Nanoparticles Synthesized via Microwave Assistance on Adsorption and Photocatalytic Degradation of Ciprofloxacin

**DOI:** 10.3390/molecules29122935

**Published:** 2024-06-20

**Authors:** Debora Briševac, Ivana Gabelica, Davor Ljubas, Arijeta Bafti, Gordana Matijašić, Lidija Ćurković

**Affiliations:** 1 Faculty of Mechanical Engineering and Naval Architecture, University of Zagreb, 10000 Zagreb, Croatia; debora.brisevac@fsb.unizg.hr (D.B.); ivana.gabelica@fsb.unizg.hr (I.G.); davor.ljubas@fsb.unizg.hr (D.L.); 2 Faculty of Chemical Engineering and Technology, University of Zagreb, 10000 Zagreb, Croatia; abafti@fkit.unizg.hr (A.B.); gmatijas@fkit.unizg.hr (G.M.)

**Keywords:** titanium dioxide, microwave-assisted synthesis, characterization, photocatalysis, ciprofloxacin

## Abstract

In this study, the optimal microwave-assisted sol-gel synthesis parameters for achieving TiO_2_ nanoparticles with the highest specific surface area and photocatalytic activity were determined. Titanium isopropoxide was used as a precursor to prepare the sol (colloidal solution) of TiO_2_. Isopropanol was used as a solvent; acetylacetone was used as a complexation moderator; and nitric acid was used as a catalyst. Four samples of titanium dioxide were synthesized from the prepared colloidal solution in a microwave reactor at a temperature of 150 °C for 30 min and at a temperature of 200 °C for 10, 20, and 30 min. The phase composition of the TiO_2_ samples was determined by X-ray diffraction analysis (XRD) and Fourier-transform infrared spectroscopy (FTIR). Nitrogen adsorption/desorption isotherms were used to determine the specific surface area and pore size distributions using the Brunauer–Emmett–Teller (BET) method. The band-gap energy values of the TiO_2_ samples were determined by diffuse reflectance spectroscopy (DRS). The distribution of Ti and O in the TiO_2_ samples was determined by SEM-EDS analysis. The effects of adsorption and photocatalytic activity of the prepared TiO_2_ samples were evaluated by the degradation of ciprofloxacin (CIP) as an emerging organic pollutant (EOP) under UV-A light (365 nm). The results of the photocatalytic activity of the synthesized TiO_2_ nanoparticles were compared to the benchmark Degussa P25 TiO_2_. Kinetic parameters of adsorption and photocatalysis were determined and analyzed. It was found that crystalline TiO_2_ nanoparticles with the highest specific surface area, the lowest energy band gap, and the highest photocatalytic degradation were the samples synthesized at 200 °C for 10 min. The results indicate that CIP degradation by all TiO_2_ samples prepared at 200 °C show a synergistic effect of adsorption and photocatalytic degradation in the removal process.

## 1. Introduction

Pharmaceuticals consist of very complex molecules that possess different biological and chemical properties. The problem today is their constant release into the environment, which causes a negative impact on nature and the living world [1,2,3]. The primary source of pollution is wastewater collected from households, hospitals, industries, etc. The result of wastewater discharge into the environment is the pollution of soil, surface, and underground waters, as well as sources of drinking water [4,5,6]. The most common pharmaceuticals in wastewater are antibiotics, hormones, β-blockers, anti-inflammatory drugs, etc. [2,4]. Ciprofloxacin (CIP) is a broad-spectrum synthetic antibiotic that belongs to the group of fluoroquinolone antibiotics used for treating both humans and animals. It is most often used for mild to moderate inflammations of the urinary and respiratory systems [3,6]. Conventional water treatment processes are not efficient enough for the degradation of complex substances such as pharmaceuticals, their metabolites, or transformation products [1]. Thus, advanced oxidation processes (AOPs), especially photocatalytic oxidation, which can be used alone but also in combination with other conventional processes, have become a newer possible solution for removing pharmaceuticals from water [7].

Titanium dioxide (TiO_2_) is a non-toxic, biocompatible, and inexpensive material with a very high dielectric constant and chemical stability. It most commonly occurs in nature in the form of anatase and rutile and rarely as brookite [8,9]. TiO_2_ is a semiconductor with a band-gap energy ranging from 3.0 eV (as rutile) to 3.2 eV (as anatase). Moreover, it is continuously studied and used as a photocatalyst due to its superior photocatalytic activity, low cost, good chemical stability, and non-toxicity. Čizmić et al. prepared nanostructured TiO_2_ film for successful degradation of azithromycin. They evaluated factors such as pH value, different water matrices, various pollutants, and radiation sources to find the optimum ones [1]. The same TiO_2_ film was deposited on a glass substrate and used for photocatalytic degradation of lissamine green B dye by Ćurković et al. [10]. Gabelica et al. prepared ternary core-shell Fe_3_O_4_/SiO_2_/TiO_2_ nanocomposite for the degradation of ciprofloxacin with the prospect of recycling and reuse [11]. However, high band-gap energy limits the photoactivation of TiO_2_ to UV light, and it has to be modified via doping with various metals, non-metals, and composites to shift its photoactivity to the visible light range [12]. Sanchez Tobon et al. prepared nitrogen-doped and reduced graphene oxide composite (N/TiO_2_/rGO) for degradation of different micropollutants, such as ciprofloxacin, diclofenac, and salicylic acid, under different radiation sources [13]. They observed a synergistic effect of adsorption and photocatalysis, while the degradation rate was affected by the radiation source, irradiation intensity, and type of pollutant. Malakootian et al. immobilized TiO_2_ nanoparticles on a glass plate and investigated the photocatalytic degradation of ciprofloxacin in an aqueous solution [14].

TiO_2_ is also used in many fields, such as the cosmetic, paper, paint, and varnish industries. Due to the large surface area of small particles in nano-sized TiO_2_, it is often used as a self-cleaning material for tiles, interior furnishings, coatings on indoor and outdoor lamps, traffic signs, and others [15].

Titanium dioxide can also be used in optical devices. Its thermo-optic properties and in situ refractive index were determined by Gülşen et al. using multimode optical fibers [16]. They concluded that the optical properties of TiO_2_ films strongly depend on the oxidation rate of films and exposure time to air. In recent years, many TiO_2_ materials with new compositions and structures have been prepared and used for various sensors [17,18]. Bai and Zhou reviewed three typical sensors: a gas sensor, a sensor for detecting soluble organics in water, and a biosensor for detecting biological substances [17]. The large surface area of nanostructured TiO_2_ particles makes them applicable for solar-cell production. Chougala et al. presented a sol-gel method for the preparation of TiO_2_ nanoparticles and their application in dye-sensitized solar cells [19].

Different methods exist for synthesizing TiO_2_, such as the sol-gel method [10], the sol-gel-hydrothermal method [20], the hydrothermal method [21], the solvothermal method [22], and the recently appeared microwave-assisted synthesis method [11,13,23].

The sol-gel synthesis is a universal process for producing various new materials, covering a broad and interdisciplinary research area ranging from nano to macro dimensions. In the typical sol-gel process, a colloidal suspension or sol is formed by the hydrolysis of precursors and their polymerization. There are many influencing factors for the sol-gel synthesis process—but critical ones are the precursor and solvent selection, pH, temperature, catalysts/additives, stoichiometry, drying, etc. Obtaining TiO_2_ by the sol-gel method involves the chemical conversion of titanium salts or solutions of organotitanium substances into monomeric titanium hydroxide (Ti(OH)_4_) and its subsequent polycondensation to form colloidal particles. Unlike other methods, the sol-gel method allows for control over the structure and morphology of TiO_2_ particles, the optimization of energy costs, and the use of simple and affordable technological equipment. However, to achieve TiO_2_ nanoparticles, the obtained gel must be heated in a kiln at high temperatures for a few hours, significantly increasing production costs [24,25].

Microwave-assisted synthesis is a technique that offers rapid and efficient material processing, especially in comparison to the classical hydrothermal or solvothermal methods. Microwave radiation is electromagnetic radiation in the frequency range from 0.3 GHz to 300 GHz, corresponding to wavelengths from 1 mm to 1 m [26]. Microwaves can be used for nanomaterial synthesis, solid-state chemistry, nanotechnology, and organic synthesis [27]. Organic reactions can be accelerated, and the selectivity of the resulting products can be enhanced by choosing appropriate microwave parameters. Microwave-assisted synthesis offers several advantages over conventional heating, such as instantaneous and rapid heating (deep internal heating), high-temperature homogeneity, and selective heating [28].

Compared to the other synthesis methods, microwave-assisted synthesis is based on the interaction of microwaves with the material at the molecular level. Specifically, molecular dipoles absorb microwaves and convert their energy into heat, resulting in heat generation within the material or suspension. The temperature gradient in microwave synthesis is opposite to that in conventional heating, i.e., it is a so-called core heating rather than heating from the outside [29]. Heat transfer by microwaves depends on the specific loss factor characteristic of each solvent. The higher this value, the better the solvent will absorb microwaves. During synthesis, a large amount of energy is released, enabling rapid heating rates, high yields, and precise heating control, ultimately ensuring early phase formation and uniform particle size distribution. The shortened synthesis time also prevents overheating phenomena. In a closed reactor, under the influence of microwaves, solvents can be heated to a temperature higher than the (atmospheric) boiling temperature without boiling [23].

Microwave dielectric heating relies on the ability of a material (such as a solvent or reagent) to absorb microwave energy and convert it into heat. Heating mechanisms by microwave energy consist of heating with the electric and magnetic fields, which are controlled by different mechanisms. There are two main mechanisms for electric field heating, dipolar polarization and ionic conductivity, while the combination of these two mechanisms is interfacial polarization. Dipolar polarization is a mechanism involving the rotation of polar molecules that align with the electric field of microwaves, where heat and friction are generated due to the continuous alignment of polar molecules while the electric field regularly oscillates. As for ionic conductivity, an electric current is generated by the oscillation of ions back and forth due to the electric force of the microwaves. This produced current encounters with internal resistance, leading to collisions between charged molecules and their neighboring molecules, resulting in material heating [30].

Traditionally, organic synthesis is conducted using conductive heating with an external heat source (e.g., oil bath or heating mantle). In addition to being relatively slow, this method is inefficient for energy transfer in the system because it relies on convection currents and the thermal conductivity of different materials through which heat must pass to reach the reaction mixture. Consequently, the reaction vessel has a higher temperature than the reaction mixture. On the other hand, microwave (MW) irradiation produces efficient internal heating (volumetric heating in the core) by directly coupling the MW energy with molecules (solvents, reagents, catalysts) present in the reaction mixture. For MW heating, reaction vessels are made of materials that are (almost) microwave-transparent and are used to allow radiation to pass through their walls. Materials used for this purpose include borosilicate glass, quartz, or Teflon. This (near) microwave passage results in a reversed temperature gradient compared to conventional heating. If the microwave cavity is well designed, the temperature rise will be uniform throughout the sample [26].

The main disadvantage of the most commonly used conventional methods (hydrothermal, solvothermal, sol-gel, precipitation, etc.) for the synthesis of TiO_2_-based photocatalysts is the long synthesis time associated with higher energy consumption. On the other hand, the microwave-assisted method has significant attractions because it is considered an environmentally friendly and highly efficient method with rapid heating at low temperatures, higher yield, and better reproducibility of nanomaterials. Therefore, this research aims to prepare titanium dioxide particles using the sol-gel method and microwave-assisted synthesis at different exposure times to MW radiation and at different temperatures. The structure of prepared materials in correlation with their photocatalytic properties was investigated.

## 2. Results and Discussion

### 2.1. Characterization of Prepared TiO_2_ Photocatalyst

**Figure 1:**
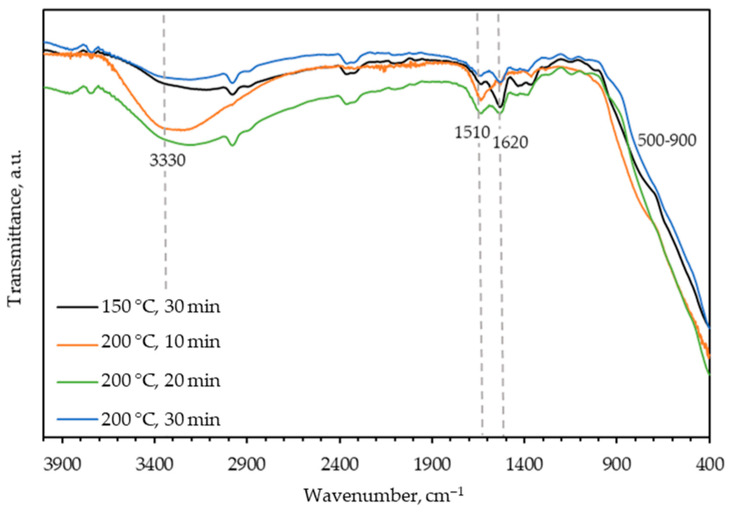
FTIR spectra of prepared TiO_2_ photocatalyst on 150 °C for 30 min (black line), 200 °C for 10 min (orange line), 200 °C for 20 min (green line) and 200 °C for 30 min (blue line).

The FTIR spectrum of the obtained TiO_2_ (Figure 1) displays characteristic Ti-O vibration and Ti-O-Ti stretching bands in the range of approximately 500 to 900 cm^−1^. At around 1500 and 1600 cm^−1^, Ti-OH vibrational stretches are observed, as well as –OH asymmetric and symmetric vibrational stretches at approximately 3300 cm^−1^. These –OH stretches may originate from adsorbed water. The obtained results are consistent with the literature [19,31,32].

**Figure 2:**
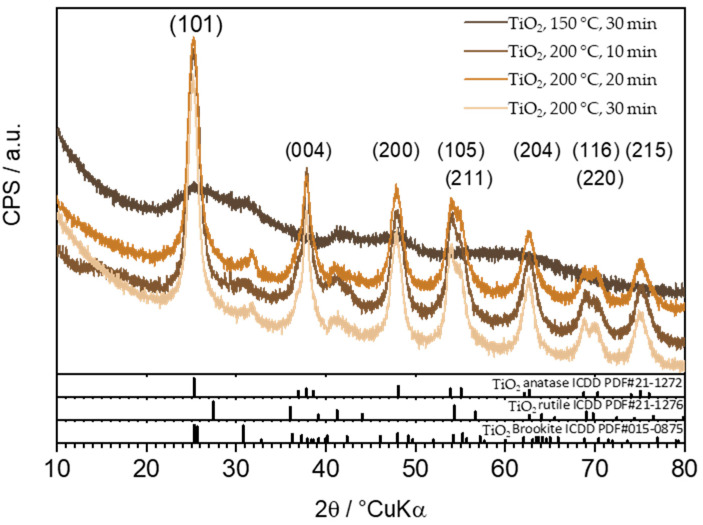
Diffractograms of prepared TiO_2_ photocatalysts on 150 °C for 30 min 200 °C for 10 min, 200 °C for 20 min and 200 °C for 30 min.

The diffractograms of the prepared TiO_2_ photocatalyst shown in Figure 2 indicate that the temperature and processing time influence the crystalline phases present in the samples. Thus, the sample processed at 150 °C shows no pronounced diffraction peaks, while samples processed at 200 °C indicate the presence of the TiO_2_ anatase phase (ICDD PDF#21-1272). Among samples treated at 200 °C for different durations, no drastic change in intensities was observed. Still, it can be noted that the intensities of samples treated for 30 min, compared to those treated for 20 and 10 min, exhibit slightly more intense and narrower diffraction peaks, suggesting an increase in the crystallite size within the sample.

**Figure 3:**
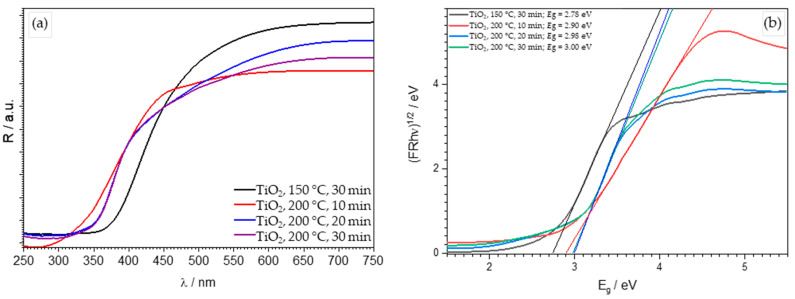
Reflectance spectra (**a**) and Tauc’s graphical representation (**b**) for prepared TiO_2_ photocatalyst at 150 °C for 30 min, 200 °C for 10 min, 200 °C for 20 min and 200 °C for 30 min.

Diffuse reflectance spectroscopy (Figure 3) indicates that an increase in processing temperature from 150 °C to 200 °C results in a shift of reflection towards shorter wavelengths (from approximately 400 to 350 nm) and, consequently, an increase in the band-gap energy from 2.78 eV to 3.0 eV, indicating an indirect transition (Table 1; Figure 3b). Figure 3a presents reflectance measurements of prepared TiO_2_ samples. The obtained values are in good agreement with the literature data for TiO_2_ rutile, suggesting the presence of defects within the sample, causing the shift in the band-gap energy (3.2 eV for anatase) [33]. The best option to verify this assumption is to use advanced analysis methods such as XPS (X-ray photoelectron spectroscopy).

**Figure 4:**
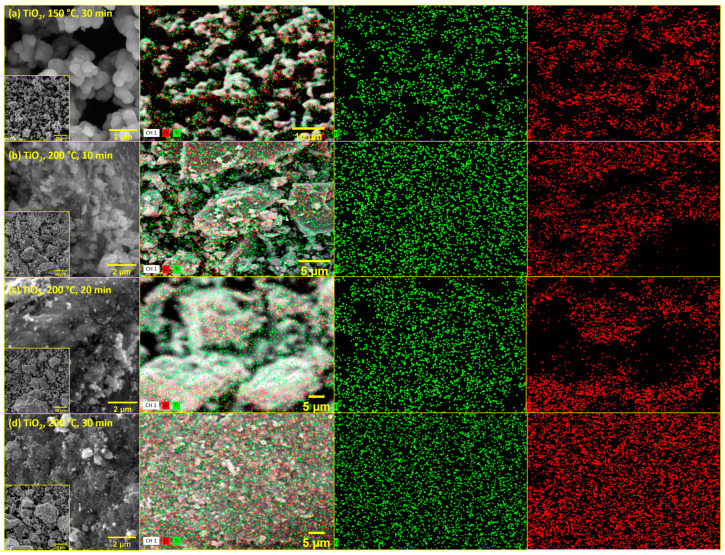
SEM-EDS mapping of prepared TiO_2_ photocatalyst at (**a**) 150 °C for 30 min, (**b**) 200 °C for 10 min, (**c**) 200 °C for 20 min, and (**d**) 200 °C for 30 min with EDS mapping (both Ti and O), and separate Ti (green) and O atoms (red).

Figure 4 presents SEM micrographs along with EDS mapping conducted for all prepared samples. Images of lower magnifications are in the marked squares in the down-left corner of the SEM micrographs. As visible, samples prepared at higher temperatures differ from those prepared at 150 °C. Smaller particles are observed for the sample prepared at 150 °C, which is in excellent correlation with the values of specific surface areas. Moreover, when discussing EDS mapping, all samples show homogeneous coverage with Ti (green) and O (red) atoms. When considering a ratio, all samples correspond to titanium dioxide.

**Figure 5:**
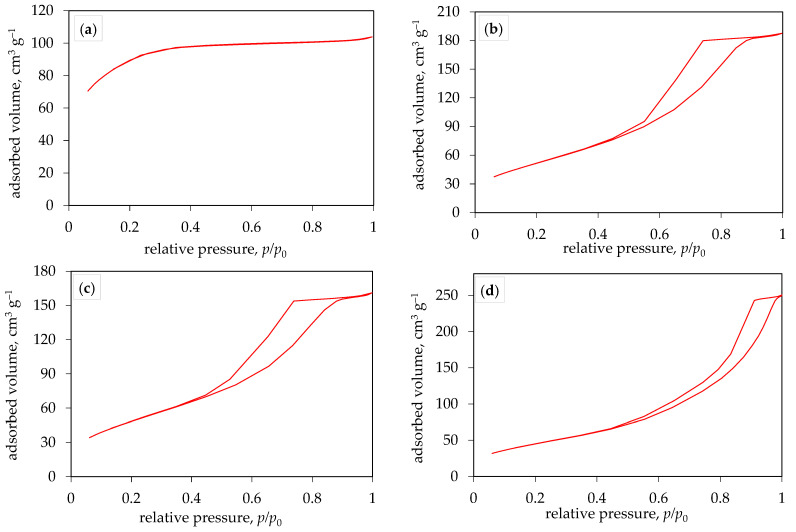
Nitrogen adsorption/desorption isotherms for prepared TiO_2_ photocatalyst at (**a**) 150 °C for 30 min, (**b**) 200 °C for 10 min, (**c**) 200 °C for 20 min, and (**d**) 200 °C for 30 min.

**Figure 6:**
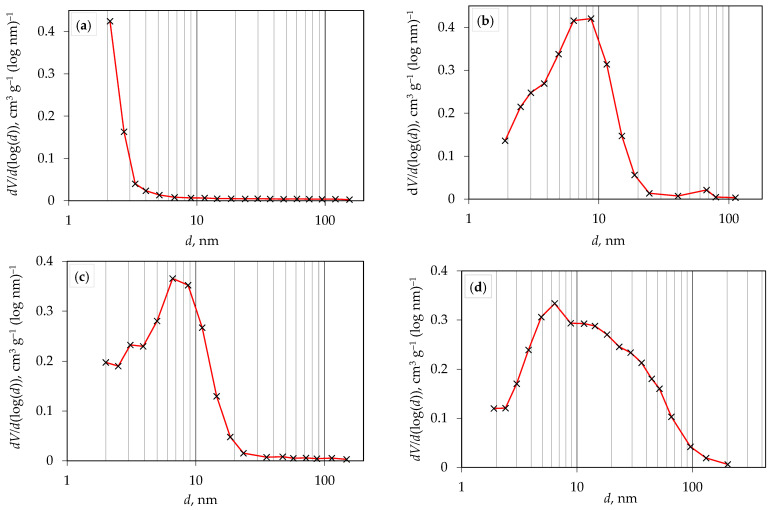
Pore size distribution for prepared TiO_2_ photocatalyst at (**a**) 150 °C for 30 min, (**b**) 200 °C for 10 min, (**c**) 200 °C for 20 min, and (**d**) 200 °C for 30 min.

Figure 5a–d shows the nitrogen adsorption/desorption isotherms of all prepared TiO_2_ photocatalysts and indicates the volume of adsorbed/desorbed nitrogen over the entire range of relative pressures (0–1). According to the new IUPAC classification [34], the nitrogen adsorption/desorption isotherms of all TiO_2_ samples synthesized at 200 °C (Figure 5b–d) correspond to type IV(a), while the isotherm obtained for the sample synthesized at 150 °C (Figure 5a) corresponds to type I(b). The hysteresis obtained in the case of a type IV(a) isotherm indicates the presence of capillary condensation, which occurs when the pore width exceeds a certain critical width, usually around 4 nm in the case of nitrogen adsorption in cylindrical pores at 77 K [35,36,37]. Type I(b) isotherms are found in materials that exhibit a certain range of wider micropores and possibly narrow mesopores (smaller than 2.5 nm), which is consistent with the pore size distribution obtained for the sample synthesized at 150 °C (Figure 6a). Figure 6a–d shows the pore size distribution of all produced TiO_2_ photocatalysts. From the results shown in Table 2, it can be concluded that the average pore diameter of the TiO_2_ samples is between 2.0 and 9.1 nm, indicating that the prepared TiO_2_ samples have a mesoporous structure with a certain amount of micropores obtained in the sample synthesized at 150 °C. This resulted in a higher specific surface area and smaller pore diameter of TiO_2_ at a lower synthesis temperature. For the samples synthesized at 200 °C, an increase in synthesis time leads to a decrease in specific surface area, which is due to a shift towards mesoporous regions, as can be seen in Figure 6b–d. Degussa P25 TiO_2_ powder consists of both anatase and rutile phases (75–80% anatase and 20–25% rutile) [38], while samples prepared in this research contain only the anatase phase.

### 2.2. Adsorption of Ciprofloxacin by TiO_2_ Nanoparticles

**Figure 7:**
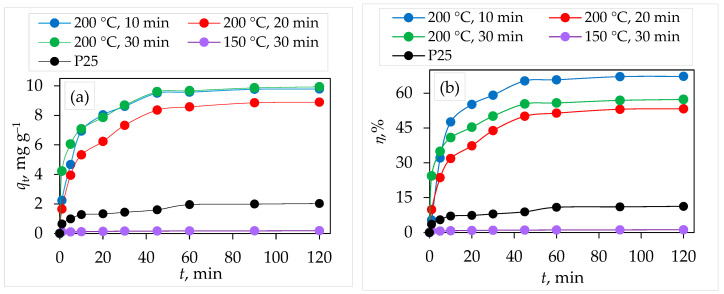
Effect of contact time and type of TiO_2_ sample on (**a**) amount adsorbed (*q*_t_, mg g^−1^) and (**b**) removal efficiency (*η*,%) of CIP, *m* (TiO_2_)=25 mg, *γ* (CIP) = 5 mg L^−1^, *V* (CIP)=100 mL, *T* = 25 °C.

Results of the amount of adsorbed CIP on different TiO_2_ nanoparticles by adsorption process are presented in Figure 7a. The removal efficiency of CIP by TiO_2_ nanoparticles is presented in Figure 7b. The results indicated that the adsorption process referred to the two following steps: the first step required a cca of 45 min, which is directly related to the gradual increase in the amount of removal values with increasing the adsorption time to reach an equilibrium condition. The second step refers to the complete saturation of the TiO_2_ nanoparticles by the sorbed CIP molecules and the reached maximum removal values (equilibrium) at 45 min of sorption time. It was found that the removal efficiency of CIP by microwave-assisted synthesized TiO_2_ nanoparticle at 150 °C for 30 min is negligible (removal efficiency at equilibrium is 1.2%). For the TiO_2_ samples synthesized at 200 °C, removal efficiency (*η*,%) of CIP increased with increasing microwave reaction time from 10 min to 30 min (Figure 6b): 10 min (67.23%) > 20 min (53.28%) > 30 min (50.57%).

**Figure 8:**
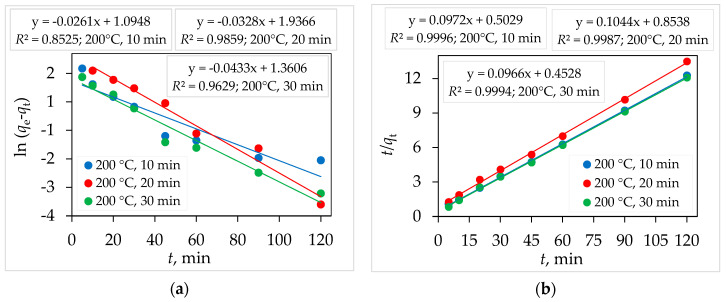
Kinetic studies of adsorption CIP onto TiO_2_ samples (**a**) pseudo-first-order and (**b**) pseudo-second-order; *m* (TiO_2_) = 25 mg, *γ* (CIP) = 5 mg L^−1^, *V* (CIP) = 100 mL, *T* = 25 °C.

The study of adsorption kinetics is important because it provides valuable insights into the adsorption process, including the required time for adsorption and the rate of CIP uptake on TiO_2_ nanoparticles. Due to the low adsorption amount of CIP on Degussa P25 and the sample synthesized at 150 °C for 30 min, the kinetics of pseudo-first- and pseudo-second-order were determined only for samples synthesized at 200 °C for 10, 20, and 30 min. The adsorption kinetics of CIP (adsorbate) on prepared TiO_2_ nanoparticles (adsorbent) was investigated by using two kinetic models: Lagergren’s pseudo-first-order (PFO, Equation (1)) and Ho’s pseudo-second-order (PSO, Equation (2)) [11]:

(1) $$\ln\left(q_{e}-q_{t}\right)=\ln q_{e}-k_{1}t$$

(2) $$\frac{t}{q_{t}}=\frac{1}{k_{2}q_{e}^{2}}+\frac{t}{q_{e}}$$

where *q*_e_ and *q*_t_ are the amounts of CIP, (mg g^−1^) removal by adsorption on TiO_2_ samples at equilibrium and at time *t* (min); *k*_1_ (min^−1^) and *k*_2_ (g mg^−1^ min^−1^) are the rate constants of the pseudo-first-order (PFO) and pseudo-second-order (PSO) reactions, respectively. The values of both kinetic model parameters (*q*_e_, *k*_1_, *k*_2_, *R*^2^) are obtained from the linear plots of ln (*q*_e_ − *q*_t_) against *t* for PFO (Figure 8a) and *t*/*q*_t_ against *t* for PSP (Figure 8b). The obtained values of the kinetic parameters of the CIP sorption by TiO_2_ samples are listed in Table 3. The pseudo-first-order model predicts that CIP would accumulate on the sorbent surface with time, while the pseudo-second-order model assumes that chemisorption is the rate-determining step [39].

The high determination coefficient (*R*^2^) values (*R*^2^ in the range of 0.9983–0.9999), as well as the agreement between experimental (*q*_e_) and calculated (*q*_cal_) values, show that CIP sorption on TiO_2_ nanoparticles follows the pseudo-second-order model (Table 3).

### 2.3. Photocatalytic Degradation Performance of TiO_2_ Nanoparticles

**Figure 9:**
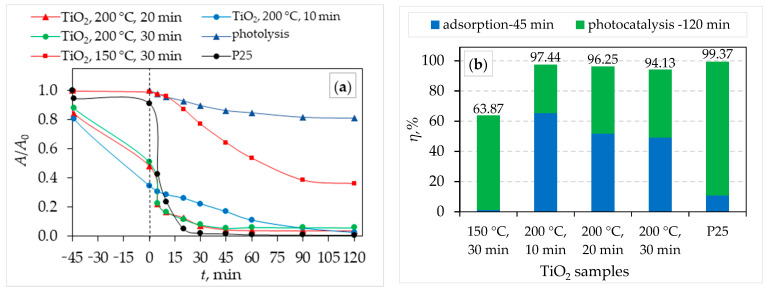
(**a**) Photocatalytic degradation of CIP by TiO_2_ samples, and (**b**) removal efficiency by synergistic effects by adsorption and photocatalysis.

A comparison of the photocatalytic activities of TiO_2_ samples synthesized via microwave assistance with commercially available TiO_2_ P25 to CIP under UVA irradiation was determined, and the results are presented in Figure 9. It can be seen that the highest overall CIP removal (photocatalytic activity+ adsorption) was achieved with P25 (with a removal efficiency of 99.37%), followed by a microwave-assisted TiO_2_ sample synthesized at 200 °C for 10 min (with a removal efficiency of 97.44%, which is very close to the removal efficiency of P25 TiO_2_). The microwave-assisted TiO_2_ sample synthesized at 150 °C for 10 min showed the lowest removal efficiency of 63.87%. All microwave-assisted TiO_2_ samples synthesized at 200 °C show synergistic effects of adsorption and photolytic removal of CIP. The kinetic constant for the pseudo-first-order model is obtained by the slope of the plot—ln (*C*/*C*_0_) versus the irradiation time *t*. Table 4 gives the list of the pseudo-first-order (*k*_1_, min^−1^) kinetic constant and corresponding determination coefficients (*R*^2^) for CIP removal by the synthesized TiO_2_ samples as well as the commercial TiO_2_ P25. The values of determination coefficients (*R*^2^ > 0.90) indicated that CIP removal follows a pseudo-first-order model.

## 3. Materials and Methods

### 3.1. Preparation of TiO_2_ Nanoparticles

**Figure 10:**
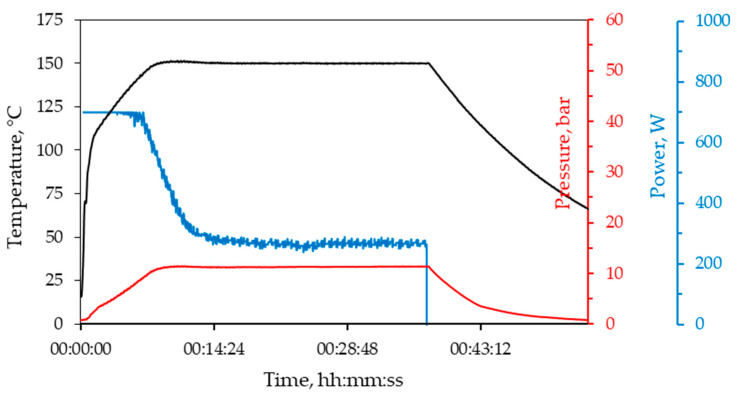
Inner pressure, temperature, and power supplied by the microwave oven during the synthesis of TiO_2_ nanoparticles at 150 °C for 30 min.

TiO_2_ nanoparticles were prepared using the microwave-assisted sol-gel method. TiO_2_ sol (colloidal solution) was prepared by mixing the following analytical grade reagents: titanium(IV) isopropoxide (TIP, Ti(OCH(CH_3_)_2_)_4_, 98%, Sigma–Aldrich, St. Louis, MO, USA) as the precursor, i-propyl alcohol (PrOH, C_3_H_7_OH, 99.9%, Gram-Mol, Zagreb, Croatia) as the solvent, acetylacetone (AcAc. CH_3_(CO)CH_2_COCH_3_, 99%+, VWR International, Radnor, PA, USA) as the chelating agent, and nitric acid (HN, HNO_3_, 65%, Carlo Erba Reagents, Val-de-Reuil, France) as the catalyst. The molar ratio of these reactants in all colloidal solutions was TIP:PrOH:AcAc:HN = 1:35:0.63:0.015 [10]. The colloidal solution is then transferred into Teflon tubes, which are sealed and placed into ceramic vessels, which are then placed in a microwave reactor (Microwave Reaction System SOLV, Multiwave PRO, Anton Paar, Graz, Austria) at 150 °C for 30 min, and 200 °C for 10, 20, and 30 min. During the microwave-assisted synthesis, inner pressure and temperature were monitored and recorded by a *p*/*T* sensor (Anton-Paar GmbH, Graz, Austria). Recorded data for the TiO_2_ sample synthesized at 150 °C for 30 min is shown in Figure 10. Changes in reaction parameters for other TiO_2_ samples are given in the Supplementary Materials (Figures S1–S3). After synthesis, the samples were rinsed with distilled water and alcohol. TiO_2_ particles were separated by a centrifuge (Centric 322A, Tehtnica, Zelezniki, Slovenia) at a speed of 2000 revolutions per minute for 5 min. The particles were then dried in a drying oven (Instrumentaria Zagreb ST-05, Instrumentaria d.d., Sesvete, Croatia) at 60 °C for 4 h. The obtained particles were then ground in a mortar and characterized using the methods described below.

### 3.2. Characterization of Prepared TiO_2_ Nanoparticles

FTIR analysis of the synthesized TiO_2_ samples was conducted using a spectrometer (IRSpirit, Shimadzu, Tokyo, Japan) equipped with an additional ATR (attenuated total reflectance). The X-ray diffraction method was used to determine the crystalline phases in the samples. XRD analysis of the samples was performed using a diffractometer (D8 Advance, Bruker, Billerica, MA, USA), with CuKα radiation at an accelerating voltage of 40 kV and a current of 25 mA. Data was collected in a Bragg–Brentano configuration in the range of 10 to 80 (or 10 to 60) 2θ angles with a step size of 0.02° 2θ and a step duration of 0.6 s. Diffuse reflectance spectroscopy was performed on a spectrophotometer (Lambda 1050+, PerkinElmer, Shelton, CT, USA) for all prepared samples. Reflectance spectra (Figure 3) were recorded in the wavelength range from 250 nm to 750 nm (UV-Vis region), with a recording resolution of 1 nm. Barium (II) sulfate (BaSO_4_) was used as the standard. The band-gap energy, *E*_g_, was then determined from the obtained spectra using the Kubelka–Munk equation or Tauc plot. Scanning electron microscopy (SEM) was carried out using a Tescan Vega Easyprobe 3 microscope (Brno, Czech Republic). Imaging was performed in secondary electron mode (SE) at an accelerating voltage of 10 kV and a working distance of about 5 mm. Nitrogen adsorption/desorption isotherms (Figure 5) of the obtained TiO_2_ samples were recorded using an ASAP-2000 instrument (Micromeritics, Norcross, GA, USA) at a temperature of −196 °C. Prior to this, potentially adsorbed substances on the samples needed to be removed by degassing at a temperature of 150 °C until a vacuum of less than 50 mm Hg was achieved. The specific surface area (*S*_BET_) of the samples was calculated using the BET model, using five points within the relative pressure range of 0 to 0.2. Pore volume (*V*_p_) and pore size distribution were calculated from the nitrogen adsorption branch of the isotherm using the Barrett–Joyner–Halenda (BJH) model. The values of specific surface area and average pore diameter of TiO_2_ samples are presented in Table 2.

The specific surface area of synthesized TiO_2_ particles (Table 2) is higher when compared to other commercial ones: 8 m^2^ g^−1^ [40], 9 m^2^ g^−1^ [41], and 50–54 m^2^ g^−1^ [38].

### 3.3. Photolytic, Adsorption, and Photocatalytic Testing

Adsorption experiments were performed in a batch reactor in the dark by continuously stirring a 25 mg TiO_2_ sample with 100 mL of CIP solution (5 mg L^−1^). Samples were taken from the batch reactor at intervals (0, 1, 5, 10, 20, 30, 45, 60, 90, and 120 min), filtered using a 0.45 µm mixed cellulose ester membrane filter, and analyzed using a UV-Vis spectrophotometer (HEWLETT PACKARD, Model HP 8430, Palo Alto, CA, USA) at 273 nm (maximum absorption peak of CIP). For all TiO_2_ samples (four synthesized samples and commercially available P25, Degussa-Hűls Co., Frankfurt, Germany), equilibrium sorption was reached after 45 min. For photocatalytic experiments, 25 mg of the TiO_2_ sample was dispersed in 100 mL of CIP solution (5 mg L^−1^) and irradiated from above with a UV-A lamp, model UVAHAND LED (Dr. Hönle AG, UV-Technologie, Gilching, Germany) (peak on 365 nm, 70 W), the distance of the lamp from the reaction mixture in reactor was 20 cm. First, the suspension was stirred for 45 min in the dark to ensure adsorption-desorption equilibrium, which was determined previously by the adsorption test. Afterward, the lamp was turned on, and the suspension was irradiated for 2 h. During the whole experiment, the temperature was kept at 25 °C by a thermostatic bath. Also, the photolytic activity of the CIP solution was tested. Photolytic testing was performed using the same procedure for photocatalysis but without adding the TiO_2_ sample. All experiments were performed in triplicate to verify the reproducibility with the data variation between three measurements no greater than 5% for all samples.

## 4. Conclusions

In this study, TiO_2_ nanoparticles were synthesized using the sol-gel method in a microwave reactor at a temperature of 150 °C for 30 min, and at a temperature of 200 °C for 10, 20, and 30 min. Based on the obtained results of the characterization of the prepared TiO_2_ samples, the following conclusions can be drawn:

- FTIR analysis confirmed the presence of the TiO_2_ anatase phase (obtained without further calcination).
- XRD analysis revealed a crystalline anatase phase was present in all samples except the one synthesized at 150 °C for 30 min, where an amorphous phase was present.
- DRS analysis determined a shift in the band-gap energy from 3.2 eV to 3.0 eV, which suggests the presence of defects within the samples.
- BET analysis determined that for the TiO_2_ sample synthesized at 200 °C for 10 min, the specific surface area was the highest at 191.6 m^2^ g^−1^. The average pore diameter of this sample was 6.1 nm, corresponding to a mesoporous structure.
- Crystalline TiO_2_ nanoparticles, without the amorphous phase, with the highest specific surface area (191.6 m^2^ g^−1^), the lowest energy band gap (2.90 eV), and the highest photocatalytic activity were obtained for the sample synthesized at 200 °C for 10 min.
- The highest overall (adsorption + photocatalytic oxidation) removal efficiency was obtained for the TiO_2_ sample synthesized at 200 °C for 10 min (with a CIP-removal efficiency of 97.44% under UVA irradiation). This performance is very close to that of commercially available TiO_2_ Degussa P25 with an overall removal efficiency of 99.37%.

## Figures and Tables

**Table 1:** The calculated values of indirect band gap for prepared TiO_2_ samples.

TiO_2_ Sample		Indirect Band Gap
*T*, °C	*t*, min	*E*_g_, eV
150	30	2.78
200	10	2.90
200	20	2.98
200	30	3.0

**Table 2:** The values of specific surface area and average pore diameter of prepared TiO_2_ samples.

TiO_2_ Sample		Specific Surface Area,	Average Pore Diameter,
*T*, °C	*t*, min	$S_{BET},\;m^{2}g^{-1}$	$d_{average},\;nm$
150	30	321.9	2.0
200	10	191.6	6.1
200	20	181.1	5.4
200	30	168.5	9.1
Degussa P25 [38]		48.1 [38]	13.7 [38]

**Table 3:** The values of kinetics parameters of the CIP adsorption by TiO_2_ samples.

TiO_2_ Sample		*q*_e,exp_, mg g^−1^	Kinetic Model					
			Pseudo-First-Order			Pseudo-Second-Order		
*T*, °C	*t*, min		*k*_1_, min^−1^	*q*_e,cal_, mg g^−1^	*R* ^2^	*k*_2_, g mg^−1^ min^−1^	*q*_e,cal_, mg g^−1^	*R* ^2^
200	10	9.89	0.0261	3.0	0.8525	0.0226	10.29	0.9996
200	20	9.20	0.0328	6.9	0.9859	0.0123	9.58	0.9987
200	30	10.15	0.0433	2.6	0.9629	0.0183	10.35	0.9994

**Table 4:** The values of the pseudo-first-order kinetic constant and determination coefficients (*R*^2^) of CIP removal by TiO_2_ samples under UV-A light.

TiO_2_ Sample		*k*_1_,	*R* ^2^
*T*, °C	*t*, min	min^−1^	
150	30	0.0116	0.9972
200	10	0.0219	0.9978
200	20	0.0411	0.9365
200	30	0.0317	0.9909
Degussa P25		0.0951	0.9106
Photolysis		0.0029	0.9968

## Data Availability

The data presented in this study are available upon reasonable request from the corresponding author.

## Supplementary Materials

The following supporting information can be downloaded at: https://www.mdpi.com/article/10.3390/molecules29122935/s1, Figure S1. Inner pressure, temperature, and power supplied by the microwave oven during the synthesis of TiO_2_ nanoparticles at 200 °C for 10 min. Figure S2. Inner pressure, temperature, and power supplied by the microwave oven during the synthesis of TiO_2_ nanoparticles at 200 °C for 20 min. Figure S3. Inner pressure, temperature, and power supplied by the microwave oven during the synthesis of TiO_2_ nanoparticles at 200 °C for 30 min.
