# Supplementary material for: Effects of TiO2 Nanoparticles Synthesized via Microwave Assistance on Adsorption and Photocatalytic Degradation of Ciprofloxacin
Source: Molecules. 2024 Jun 20;29(12):2935. doi: 10.3390/molecules29122935 (PMC11207004; doi:10.3390/molecules29122935)
Supplement: Supplementary file 1 [file molecules-29-02935-s001.zip › molecules-3019731-supplementary.pdf]

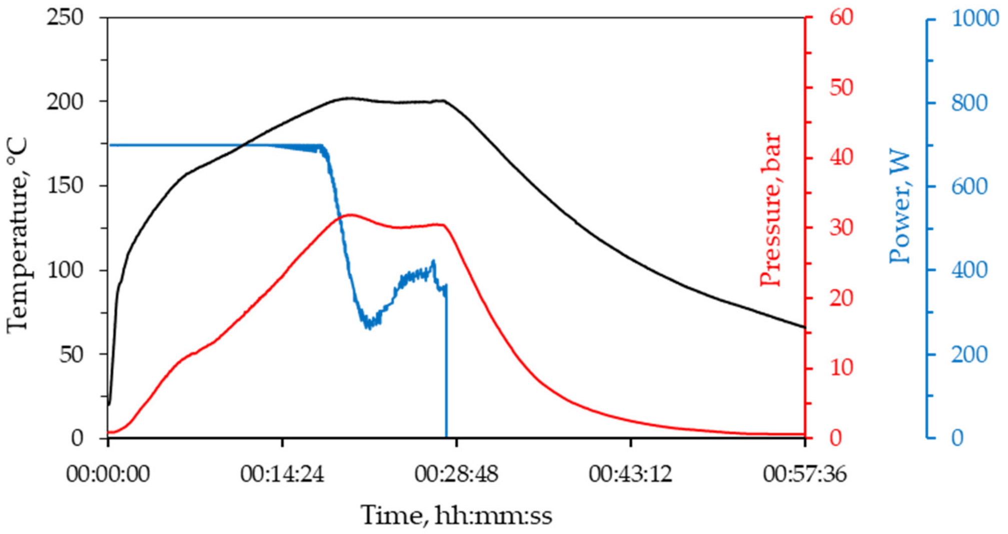

Figure S1. Inner pressure, temperature, and power supplied by the microwave oven during the synthesis of TiO_2 nanoparticles at 200°C for 10 minutes.

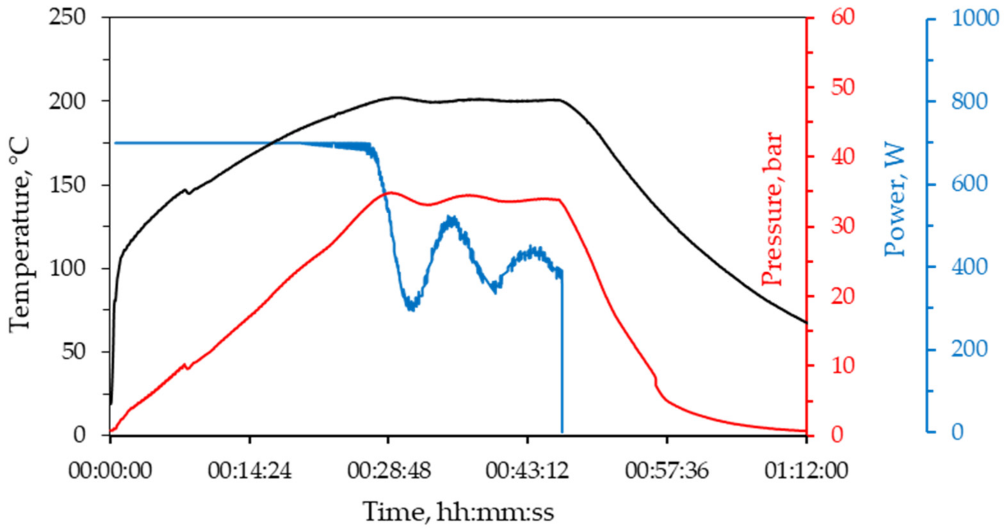

Figure S2. Inner pressure, temperature, and power supplied by the microwave oven during the synthesis of TiO_2 nanoparticles at 200°C for 20 minutes.

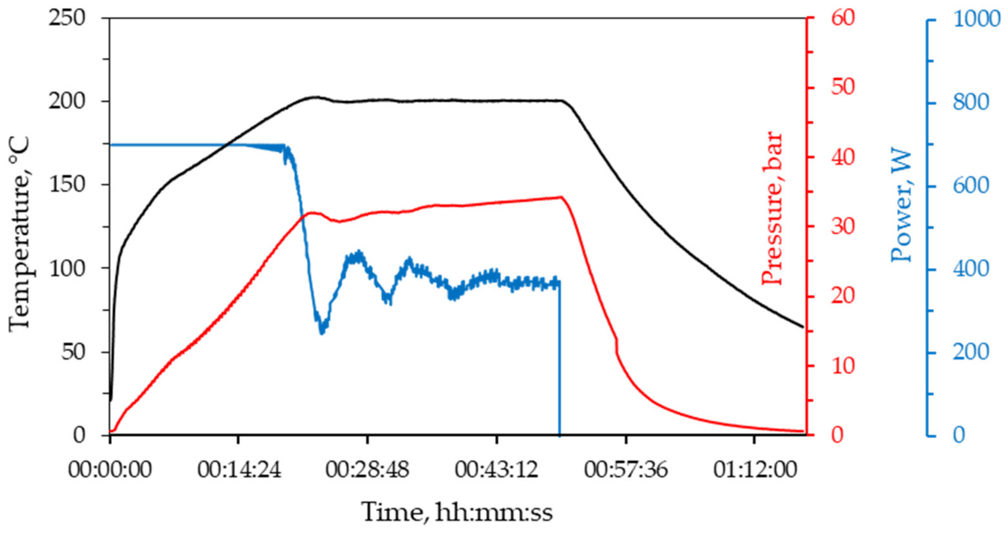

Figure S3. Inner pressure, temperature, and power supplied by the microwave oven during the synthesis of TiO_2 nanoparticles at 200°C for 30 minutes.
